# Two-Phase Dibromocyclopropanation of Unsaturated Alcohols Using Flow Chemistry

**DOI:** 10.3390/molecules25102364

**Published:** 2020-05-19

**Authors:** Runa Berg Østby, Terje Didriksen, Simen Gjelseth Antonsen, Steinar Sollien Nicolaisen, Yngve Stenstrøm

**Affiliations:** 1 Faculty of Engineering, Østfold University College, P.O. Box 700, NO-1757 Halden, Norway; 2 SINTEF Industry, P.O. Box 124 Blindern, NO-0314 Oslo, Norway; terje.didriksen@sintef.no; 3 Department of Chemistry, Biotechnology and Food Science, Norwegian University of Life Sciences, P.O. Box 5003, NO-1432 Ås, Norway; simen.antonsen@nmbu.no (S.G.A.); steinar.sollien.nicolaisen@nmbu.no (S.S.N.)

**Keywords:** dibromocyclopropanation, CHBr_3_, Makosza reaction, phase-transfer catalysis, flow chemistry, unsaturated alcohols

## Abstract

Dibromocyclopropanations are conventionally done by addition of dibromocarbene to alkenes under phase-transfer conditions in batch reactions using a strong base (50% NaOH (aq)), vigorous stirring and long reaction times. We have shown that cyclopropanation of unsaturated alcohols can be done under ambient conditions using continuous flow chemistry with 40% (*w*/*w*) NaOH (aq) as the base. The reactions were generally rapid; the yields were comparable to yields reported in the literature for the conventional batch reaction

## 1. Introduction

*gem*-Dihalocyclopropanes are important substrates in organic synthesis and have been used as versatile intermediates for the syntheses of other interesting compounds like allenes [1,2], cumulenes [3], cyclopentadienes [4,5], cyclic acetals [6,7], and also for the synthesis of natural products [8,9,10].

Traditionally, *gem*-dihalocyclopropanes were made by the Doering-Hoffman reaction [11], in which dihalocarbene is generated from haloform and *tert*-butoxide in a non-polar solvent. One of the disadvantages of the Doering-Hoffman reaction is its high sensitivity to water, often reducing the yields significantly.

A two-phase dihalocyclopropanation reaction, achieved by vigorous stirring of a solution of the starting alkene and haloform (CHX_3_, X = Cl, Br), with a concentrated (50% (*w*/*w*)) aqueous solution of sodium hydroxide, and a phase-transfer catalyst, partially solved this problem when published a decade later [12]. The discovery was important, as it was previously assumed that carbenes could not be formed in aqueous media. This reaction, known as the Makosza reaction, has been established as one of the most convenient methods for the synthesis of *gem-*dihalocyclopropanes [8,10,13].

Using unsaturated alcohols as substrates under Makosza conditions, the outcome of the reaction depends strongly on the structure of the alcohol and the precise conditions used, as the hydroxyl group/alkoxy anion may compete with the double bond for the dihalocarbene [8] and the yields of dihalocyclopropyl alcohols vary from excellent to low.

When Kleveland et al. [14] used the allylic alcohols linalool and geraniol as substrates in the Makosza reaction, they observed a surprising difference in the outcome of the reaction for the two alcohols. Linalool gave a rapid and regioselective reaction resulting in an excellent yield of the dihaloocyclopropane monoadducts, 5-(2,2-dichloro-3,3-dimethylcyclopropyl)-3-methyl-1-penten-3-ol (89%), and 5-(2,2-dibromo-3,3-dimethylcyclopropyl)-3-methyl-1-penten-3-ol (93%), while geraniol (with dichlorocarbene) gave a low yield of a mixture containing at least six components that partially decomposed under the attempted separations. Kleveland et al. [14] suggested that the difference in reactivity between linalool and geraniol is due to the primary allylic hydroxyl group competing for the dihalocarbene, and that this primary hydroxyl group has a retarding effect on the rate of addition of dihalocarbene. To alleviate the detrimental effect of the hydroxyl group, this group it is often protected either as an acetal [8,15] or an ether [8,16] during dihalocyclopropanation of unsaturated alcohols.

In the traditional Makosza reaction, vigorous stirring is essential in order to obtain a large interface area between the two immiscible liquid phases, which is needed for the mass transfer between the two phases, catalyzed by the phase-transfer catalyst. Stirring speed is thus an important factor for both reaction rate, conversion, and yield [17,18,19].

Apart from vigorous stirring, intensification of mass transfer can also be obtained in capillary-microreactors [20]. Capillary microreactors, being a special type of continuous flow reactors, offer the benefits associated with microreactors/continuous flow reactors, such as increased control of reaction temperature and time, and thus increased selectivity [21,22,23,24,25].

In the capillary microreactors, reagent solutions are usually pumped into a simple T or Y mixer, and then led through a length of tube with a typical diameter of 100–1000 μm. The high surface-to-volume ratio and the small diameter of the tube result in rapid mass and heat transfer. By immersing the tube in a temperature-controlled bath, precise control of the reaction temperature can be achieved. In addition, the reaction time, determined by the volume of the tubing and the reagent flow, is not affected by the long time used for the addition of the reagents as is the case in batch reactors.

**Figure 1:**
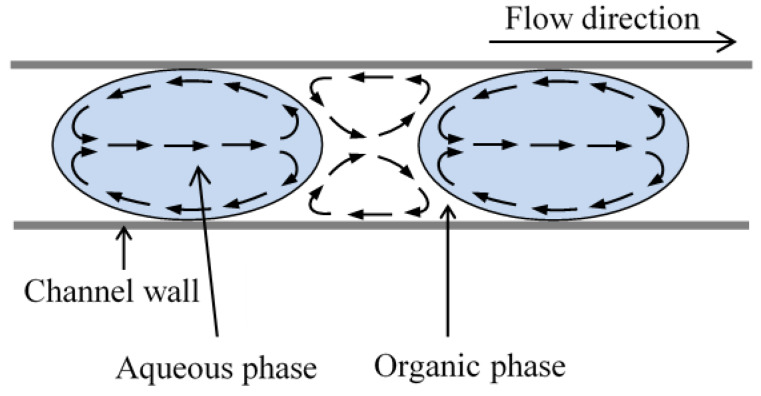
Internal circulation in alternating slugs of two immiscible liquids in a microchannel enhances mass transfer within and between the slugs. Internal flow is shown relative to the bulk velocity.

When two immiscible liquids flow through a narrow tube, they often form alternating slugs of the two liquids [26]. Due to the velocity dispersion of the liquid flow, where the velocity is highest in the center, and zero at the walls, internal circulation occurs within the liquid slugs [27] as illustrated in Figure 1. This effect results in a good mass transfer, enabling reaction rates comparable to those obtained in batch reactions, even for two-phase liquid-liquid reactions usually requiring vigorous stirring. Slug-flow reactors have been successfully used, e.g., for nitration of aromates [20], arylation of arylbromides [28], and Wittig reactions [29].

Previously, we have shown [30] that flow chemistry in a capillary microreactor can be a feasible alternative to batch chemistry for the Makosza reaction. Similarly, von Keutz et al. achieved *gem*-dichlorocyclopropanation of alkenes using packed bed flow reactors [31].

**Figure 2:**
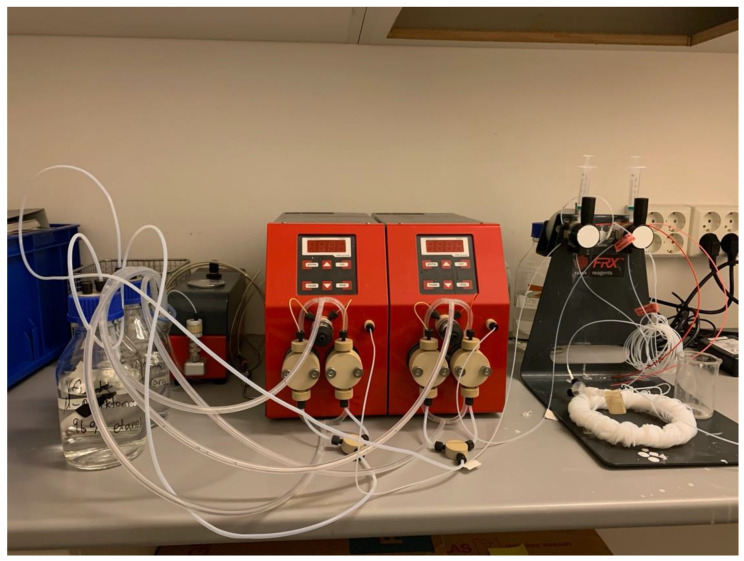
Modified flow chemistry toolkit FRX200 from Syrris Ltd.

In our case, moderate to excellent yields of dibromocyclopropanes were obtained in short reaction times from e.g., disubstituted alkenes. This encouraged us to use the same reactor system on unsaturated alcohols to see whether it would be possible to omit the protection/deprotection steps, and in addition, to get an indication of the obtainable yields when the hydroxy group is left unprotected. The setup for our experiments is shown in Figure 2.

## 2. Results and Discussion

**Scheme 1:**
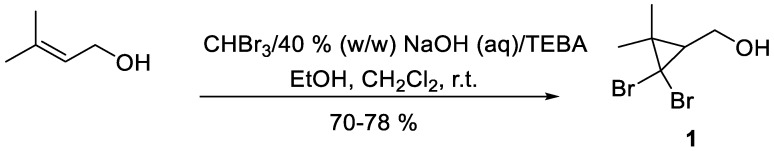
Dibromocyclopropanation of 3-methyl-2-buten-1-ol under phase-trasfer catalyst (PTC) conditions.

The unsaturated alcohols selected for testing under Makosza conditions are shown in Table 1. Benzyltriethylammonium chloride (TEBA) was used as the phase-transfer catalyst, and bromoform was the dibromocarbene-precursor, as shown for 3-methyl-2-buten-1-ol in Scheme 1.

In the traditional two-phase system, a 50% (*w*/*w*) solution of NaOH is used [8,9,10]. However, during initial experiments, we observed clogging of the capillary systems due to the viscosity of the NaOH solution. This problem was solved by reducing the base concentration to 40% (*w*/*w*) [30]. Other bases, both potassium hydroxide and lithium hydroxide were also tested without improving the yields. For both LiOH and KOH, the yields were significantly lower, and with LiOH we experienced severe clogging of the capillary tubes. Using a ratio of alkene:bromoform:TEBA of 1:1.5:0.044 together with 40% (*w*/*w*) NaOH solution in an aqueous-to-organic flow ratio (AO ratio) of 4, good to excellent yields of dibromocyclopropanes could be obtained [30].

Employing the same conditions to 3-methyl-2-buten-1-ol gave a yield of 70% of the corresponding dibromocyclopropane 1 (Table 1, entry 1). By increasing the amount of bromoform from 1.5 equivalents, the yield could be increased to 74% (using 2 equivalents), and 78% (using 2.5 equivalents). (Table 1, entries 2–3). For subsequent experiments a 1:2 or 1:2.5 ratio of alkene to bromoform was used. Several unsaturated alcohols were subjected to these conditions.

When the tertiary dienol linalool was subjected to our flow chemistry conditions, regioselective addition to the trisubstituted double bond occurred, and the dibromocyclopropane **2** was obtained in 89% yield. (Table 1, entry 4). The primary dienol geraniol, however, yielded a mixture of several products, according to ^1^H NMR and ^13^C NMR data, (Table 1, entry 5) as was also observed by Kleveland et al. [14] with dichlorocarbene. No attempts were made to separate the complex mixture, and since our observations were in accordance with the previously reported results, we did not study the reaction of geraniol with dihalocarbenes any further.

Intrigued by this result, we subjected several other alcohols to this reaction. From citronellol, that only differ structurally from geraniol by the absence of the allylic double bond, the dibromocyclopropane **4** was obtained as a mixture of diastereomers (approximately 1:1) in 57% yield when 2.5 equivalents of bromoform (compared to alkene) were used (Table 1, Entry 6). With only two equivalents, the reaction did not go to completion. 

**Scheme 2:**
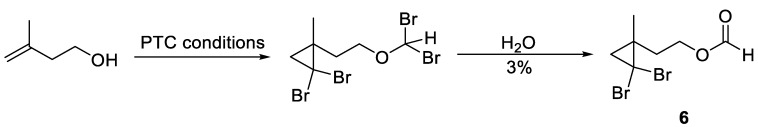
Insertion of dibromocarbene at the O-H bond and subsequent hydrolysis.

When 3-methyl-3-buten-1-ol was used as the substrate, the dibromocyclopropyl alcohol **5** was obtained in moderate yield (47%) after chromatography (Table 1, entry 7). In addition, a small amount of the formate ester **6** (3%) was isolated. The presence of the formate ester can be explained by insertion of dibromocarbene at the O-H bond and subsequent hydrolysis (Scheme 2).

The secondary alcohol, 6-methyl-5-hepten-2-ol, gave a good yield of the dibromocarbene adduct **7** as a mixture of diastereomers (approximately 1:1) (Table 1, entry 8).

When the tertiary alcohol, 2-methyl-3-buten-2-ol, was used as a substrate only small amounts of the starting material could be seen (Table 1, entry 9). This is in accordance with the literature where this alcohol has been reported to react sluggishly when subjected to dichlorocarbene under phase-transfer conditions [14].

Kleveland et al. [14] reported a 45% yield of the dibromocarbene adduct **9** as a mixture of diastereomers (1:1) when *(E)*-2-methyl-2-buten-1-ol was used as the starting material. Under our flow chemistry conditions, only one diastereomer was detected and the adduct **9** was obtained in 62% yield (Table 1, entry 10).

The secondary alcohol, 3-methyl-3-buten-2-ol (Table 1, entry 11), yielded a dibromocarbene adduct **10** as a mixture of diastereomers (2:3) according to ^1^H NMR. Purification of the mixture through a plug of silica yielded the dibromocarbene adduct **10** (49%) as a mixture of diastereomers (1:3).

In order to further investigate the scope and limitations, we tested the reaction conditions on some non-terpene-like compounds. First, both *(Z)-*and *(E)*-dec-4-en-1-ol were tested (Table 1, entries 12 and 13). The reactions were not successful, as we recovered most of the starting material in both cases. However, for both the geometric isomers, just below 10% of the material converted into a complex, inseparable mixture.

Next, we turned to styrene derived alcohols (Table 1, entries 14–19). In the experiments with cinnamyl alcohol, only pure starting material was recovered. Nishii et al. [35] have prepared the dihalocyclopropane product via a Makosza reaction. However, they protected the hydroxyl group as the corresponding tetrahydropyran. The use of protection groups has been shown to increase the yield for this type of compounds, but the styrene derived compounds are still known to be low yielding under these conditions [36]. As control experiments, we did reactions with cinnamyl alcohol protected as the corresponding tetrahydropyran and also as the *tert*-butylether, *(E)*-(3-(*tert*-butoxy)prop-1-en-1-yl)benzene (Table 1, entries 15 and 16). In both cases, only unreacted starting material was isolated. This can possibly be explained by the reaction time and the reaction temperature used in our system, as Nishii et al. used both a higher temperature and longer reaction times for similar compounds. Nishii et al. also used a 50% (*w*/*w*) NaOH (aq) solution, which, as previously mentioned, does not work well with the narrow tubes in the microreactor. Even when doubling the reaction times, our attempts were unsuccessful.

Increasing the reaction temperature above the normal boiling point of dichloromethane would require using a back-pressure regulator to pressurize the flow reactor. Our experience is that this is not feasible due to an increased propensity for clogging of the reactor system.

As Nishii et al. had success with trisubstituted alkenes [35], *(E)*-3-phenylbut-2-en-1-ol was tested in the reaction (Table 1, Entry 17). The desired product was obtained in 41% yield, confirming a higher reactivity of a trisubstituted cinnamyl alcohol compared to the disubstituted one (Table 1, entry 14).

In order to investigate whether the aromatic portion of the molecule affected the reactivity, *(E)*-3-(3,4,5-trimethoxyphenyl)prop-2-en-1-ol (Table 1, entry 18) was reacted under the same conditions, but also for this substrate we isolated only starting material. The same occurred with the corresponding ester, methyl (*E*)-3-(3,4,5-trimethoxyphenyl)acrylate (Table 1, Entry 19).

In conclusion, flow chemistry was successfully used for dibromocyclopropantion of several unsaturated alcohols under phase-transfer catalysis (PTC) using 40% (*w*/*w*) NaOH (aq) as the base. Moderate to excellent yields were achieved in less time than for batch chemistry, depending on the structure of the alcohol. The trisubstituted alkenes (Table 1, entries 1–6, 8, 10, 17) generally gave better yields than the *gem*-disubstituted (Table 1, entries 7 and 11) and monosubstituted alkenes (Table 1, entry 9). This has been explained as resulting from the increased nucleophilicity of the trisubstituted double bonds compared to di- and monosubstituted double bonds when the substituents are electron donating [8,37,38]. Our flow chemistry conditions were unsuccessful for the 1,2-disubstituted alkenes, including the aromatic ones (Table 1, entries 12–16, 18–19). This is in accordance with the literature [37]. For the successful reactions, yields comparable to the ones reported from ordinary batch-reactions were achieved. Thus, the use of microreactor technology should be an interesting alternative for the Makosza reaction (compared to the traditional batch chemistry) giving access to the general benefits of flow chemistry. The problems with the high viscosity of the concentrated base solutions and the narrow temperature window are clear limitations of this method.

## 3. Experimental Section

### 3.1. General Information

All chemicals were purchased from commercial suppliers and used without further purification unless otherwise stated. Preparation of 3-methyl-3-buten-2-ol [39], *(E)*-2-methyl-2-buten-1-ol [40], 2-(cinnamyloxy)tetrahydro-2*H*-pyran [41], *(E)*-(3-(*tert*-butoxy)prop-1-en-1-yl)benzene [42,43], *(E)*-3-phenylbut-2-en-1-ol [44], and *(E)*-3-(3,4,5-trimethoxy-phenyl)-prop-2-en-1-ol [45] was done as described in the literature. The spectral data were in accord with those reported.

Analytical thin layer chromatography (TLC) was performed on Merck DC-Alufolien Kieselgel 60 F254. Compounds were visualized by KMnO_4_ stain. Flash column chromatography was performed on silica gel (Merck Kieselgel 60, (0.040–0.063 mm, 230–400 Mesh ASTM) or VWR Chemicals/BDH Prolabo Normasil 60 (40–63 µm). In order to degas the dichloromethane, it was sonicated for 15 min prior to use in the flow system. Mass Spectrometry was performed on an Autospec Ultima (Micromass Ltd. Manchester, England) using electron ionization (EI, 70 eV).

NMR spectra were recorded on a Bruker AscendTM 400 using CDCl_3_ as a solvent and TMS as a reference. ^1^H NMR spectra were recorded at 400 MHz, and ^13^C NMR spectra were recorded at 100 MHz. IR spectra were recorded on a Perkin-Elmer Spectrum BX series FT-IR spectrophotometer using a ZnSe HATR cell (Horizontal Attenuated Total Reflectance). The flow instrumentation apparatus used was the flow chemistry toolkit FRX200 from Syrris Ltd. fitted with 2 Frx200 pumps, a reagent module containing one Syrris sample loop (PTFE, 5 mL, 0.5 mm i.d.) and one 1.0 mL sample loop (PTFE, 0.5 mm i.d.), a Y-mixer (Tube Reactor 3 input Adaptor (PCTFE) from Syrris Ltd.), and a 25 mL tube reactor (PTFE, 0.8 mm i.d.).

### 3.2. Representative Procedure, Synthesis of (2,2-Dibromo-3,3-Dimethylcyclopropyl)Methanol *(**1**)*

A 1.0 mL sample loop (PTFE) containing a solution of 3-methyl-2-buten-1-ol (1.43 mmol), CHBr_3_ (2.86 mmol), 4.3 mol% TEBA (relative to the alkene) and 0.6 vol.% ethanol (absolute) in CH_2_Cl_2_, and a 5 mL sample loop (PTFE, Syrris Ltd.) containing 40% (*w*/*w*) NaOH (aq) solution, was used. The filling of the 40% (*w*/*w*) NaOH (aq) solution into the sample loop was done very slowly and with great care, due to the high viscosity of the strongly basic NaOH solution and danger of spillage due to pressure build-up. The two solutions were simultaneously introduced into the flow system at a total flow rate of 0.50 mL/min (flow rate NaOH (aq): 0.40 mL/min, flow rate organic solution: 0.10 mL/min) at room temperature, i.e., a residence time of 50 min and an AO flow ratio of 4. The mixture was fed into brine (50 mL), and the flow was collected for 77 min at this flow rate, and then for 4 min at 2 × 1.5 mL/min (to flush the system). The pressure in the system was 1–4 bar. The reaction mixture was extracted with ethyl acetate (100 mL + 3 × 50 mL), and the combined organic phases were washed with brine (2 × 50 mL), dried (MgSO_4_), filtered and concentrated in vacuo. The residue was purified by filtering it through a plug made of 0.5 cm silica and 0.5 cm Celite 545 coarse (calcined) using ethyl acetate as the eluent. Concentration in vacuo yielded a mixture (0.31 g) containing (2,2-dibromo-3,3-dimethylcyclopropyl)-methanol (**1**): 3-methyl-2-buten-1-ol: bromoform; 88:2:10 according to ^1^H NMR. Estimated yield of **1**: 0.27 g, 74%, corresponding to a space time yield of ~0.25 moL L^−1^ h^−1^. The spectral data were in accordance with the literature [46].

### 3.3. Synthesis of 5-(2,2-Dibromo-3,3-Dimethylcyclopropyl)-3-Methyl-1-Penten-3-Ol *(**2**)*

Yield: 0.48 g of a mixture containing the dibromide **2**: linalool: bromoform; 87:9:4, according to ^1^H and ^13^C NMR. Estimated yield of the dibromide **2**, 0.42 g, 89%. The spectral data were in accordance with the literature [14].

### 3.4. Synthesis of 5-(2,2-Dibromo-3,3-Dimethylcyclopropyl)-3-Methylpentan-1-Ol *(**4**)*

2.5. equivalents of CHBr_3_ per equivalent of 3,7-dimethyl-6-octen-1-ol were used. The crude mixture was purified by column chromatography (silica, hexane: ethyl acetate; 80:20) yielding the dibromide **4** (0.27 g, 57%) as a mixture of diastereomers (approximately 1:1), according to ^1^H and ^13^C NMR. IR (HATR) ν_max_: 3338 (br, s), 2954 (s), 2926 (s), 2870 (s), 1456 (s), 1375 (s), 1147 (m), 1109 (m), 1060 (s, shoulder), 1006 (m), 963 (m), 756 (s), 740 (s) cm^−1^; ^1^H NMR (400 MHz, CDCl_3_) δ 0.89 (d, *J* = 6.5 Hz, 3H), 1.07–1.52 (m, 6H), 1.14 (s, 3H), 1.34 (s, 3H), 1.52–1.65 (m, 2H), 1.75 (br s, 1H), 3.57–3.72 (m, 2H); ^13^C NMR (100 MHz, CDCl_3_) δ 19.2 and 19.3 (CH_3_), 19.5 and 19.6 (CH_3_), 25.4 and 25.5 (CH_2_), 27.4 (CH_3_), 27.9 and 28.0 (C), 29.3 and 29.4 (CH), 35.5 and 35.6 (CH_2_), 39.6 (CH_2_), 40.01 and 40.05 (CH), 48.3 and 48.4 (C), 60.9 (CH_2_). MS, *m/z* (%) = 246 (M-HBr, 1)/248 (M-HBr, 1), 228 (10)/230 (10), 167 (40), 163 (34), 162 (33), 149 (28), 107 (13)/109 (14), 93 (24)/95 (23), 83 (100), 81 (56), 69 (74) and 67 (49). HRMS (EI^+^) *m*/*z* 325.9878. (calcd for C_11_H_20_O^79^Br_2_, 325.9881).

### 3.5. Synthesis of 2-(2,2-Dibromo-1-Methylcyclopropyl)Ethan-1-Ol *(**5**)* and 2-(2,2-Dibromo-1-Methylcyclopropyl)Ethyl Formate *(**6**)*

The crude mixture was purified by column chromatography (silica, pentane: ethyl acetate; 85:15) yielding the dibromide **5** (0.17 g, 47%) and 2-(2,2-dibromo-1-methylcyclopropyl)ethyl formate **(6)** (0.01 g, 3%), both as oils. The spectral data for the dibromoalcohol **5** were in accordance with literature [33]. *2-(2,2-Dibromo-1-methylcyclopropyl)ethyl formate (**6**).* IR (HATR) ν_max_: 2963 (s), 2928 (s), 2873 (m), 1725 (s), 1454 (m), 1430 (m), 1383 (m), 1260 (m), 1167 (s), 733 (s), 694 (s) cm^-1^; ^1^H NMR (400 MHz, CDCl_3_) δ 1.40 (s, 3H), 1.44 (d, *J* = 7.5 Hz, 1H), 1.51 (d, *J* = 7.5 Hz, 1H), 1.92–2.13 (m, 2H), 4.36 (t, *J* = 7.0 Hz, 2H), 8.05 (s, 1H); ^13^C NMR (100 MHz, CDCl_3_) δ 22.6 (CH_3_), 27.4 (C), 34.5 (CH_2_), 36.9 (CH_2_), 37.7 (C), 61.3 (CH_2_), 160.9 (CH). MS, *m/z* (%) = 238 (M-HCOOH, 14)/240 (M-HCOOH, 29)/242 (M-HCOOH, 14), 211 (10)/213 (18)/215 (9), 159 (72)/161 (70), 131 (11)/133 (12), 119 (4)/121 (3), 80 (100) and 79 (87). HRMS (EI^+^) *m*/*z* 237.8994 (calcd for C_6_H_8_^79^Br_2_, 237.8993).

### 3.6. Synthesis of 4-(2,2-Dibromo-3,3-Dimethylcyclopropyl)Butan-2-Ol *(**7**)*

The crude product was purified by column chromatography (silica, pentane: ethyl acetate; 80:20), yielding the dibromide **7** as a mixture of diastereomers (approximately 1:1, according to ^1^H and ^13^C NMR) as an oil (0.33 g, 77%). IR (HATR) ν_max_: 3343 (br, s), 2962 (s), 2926 (s), 2868 (s), 1456 (s), 1374 (s), 1335 (m), 1308 (m), 1128 (s, shoulder), 1090 (s), 773 (s), 745 (s) cm^-1^; ^1^H NMR (400 MHz, CDCl_3_) δ 1.16 and 1.17 (s, 3H), 1.20 (d, *J* = 6.2 Hz, 3H), 1.35 (s, 3H), 1.58 (s, 1H), 1.15–1.75 (m, 5H), 3.75–3.87 (m, 1H); ^13^C NMR (100 MHz, CDCl_3_) δ 19.3 (CH_3_), 23.7 (CH_3_), 24.2 and 24.4 (CH_2_), 27.42 and 27.44 (CH_3_), 28.0 and 28.1 (C), 37.6 and 37.8 (CH_2_), 39.7 and 39.9 (CH), 48.0 and 48.3 (C), 67.6 and 67.7 (CH). MS, *m/z* (%) = 280 (M-H_2_O, 18)/282 (M-H_2_O, 34)/284 (M-H_2_O, 16), 238 (3)/240 (5)/242 (2), 173 (37)/175 (35), 159 (4)/161 (4), 122 (40), 121 (83), 107 (46), 94 (100), 79 (53) and 77 (40). HRMS (EI^+^) *m*/*z* 279.9461 (calcd for C_9_H_14_^79^Br_2_, 279.9462).

### 3.7. Synthesis of (2,2-Dibromo-1,3-Dimethylcyclopropyl)Methanol *(**9**)*

Yield: a mixture (0.25 g) containing (2,2-dibromo-1,3-dimethylcyclopropyl)methanol (**9**): *(E)*-2-methyl-2-buten-1-ol: ethyl acetate: bromoform; 87:3:2:8 according to ^1^H NMR. Estimated yield of **9**: 0.22 g, 62%. The spectral data were in accordance with the literature [14]. ^1^H NMR (400 MHz, CDCl_3_) δ 1.11 (d, *J* = 6.4 Hz, 3H), 1.25 (s, 3H), 1.49 (q, *J* = 6.5 Hz, 1H), 1.85–1.98 (m, 1H), 3.63 (d, *J* = 11.9 Hz, 1H), 3.80 (d, *J* = 11.9 Hz, 1H); ^13^C NMR (100 MHz, CDCl_3_) δ 11.6 (CH_3_), 14.6 (CH_3_), 31.8 (CH), 33.2 (C), 45.4 (C), 71.3 (CH_2_).

### 3.8. Synthesis of 1-(2,2-Dibromo-1-Methylcyclopropyl)Ethanol *(**10**)*

The crude product (containing a mixture of diastereomers 2:3, according to ^1^H NMR) was purified by filtration through a small plug of silica/Celite 545 coarse (calcined), using hexane, then hexane: ethyl acetate (9:1), as eluents. The dibromide **10** was obtained as a mixture of diastereomers (approximately 1:3, according to ^1^H and ^13^C NMR) as an oil (0.18 g, 49%). The spectral data were in accordance with the literature [14]. Major isomer: ^1^H NMR (400 MHz, CDCl_3_) δ 1.32–1.37 (m, 3H), 1.35 (s, 3H), 1.46 (d, *J* = 7.4 Hz, 1H), 1.64 (d, *J* = 7.4 Hz, 1H), 1.60–1.75 (m, 1H), 3.69 (q, *J* = 6.3 Hz, 1H); ^13^C NMR (100 MHz, CDCl_3_) δ 16.7 (CH_3_), 19.1 (CH_3_), 33.3 (C), 34.6 (CH_2_), 35.0 (C), 73.9 (CH). Minor isomer: ^1^H NMR (400 MHz, CDCl_3_) δ 1.27 (d, *J* = 6.5 Hz, 3H), 1.38 (s, 3H), 1.41 (d, *J* = 7.6 Hz, 1H), 1.47 (d, *J* = 7.4 Hz, 1H), 3.77 (q, *J* = 6.5 Hz, 1H); ^13^C NMR (100 MHz, CDCl_3_) δ 15.6 (CH_3_), 19.2 (CH_3_), 32.7 (CH_2_), 34.2 (C), 38.1 (C), 73.7 (CH).

### 3.9. Synthesis of (2,2-Dibromo-3-Methyl-3-Phenylcyclopropyl)Methanol *(**16**)*

The crude product was purified by filtration through a small plug of silica/celite 545 coarse (calcined), using hexane, then hexane: ethyl acetate (9:1), as eluents to yield compound **16** as an oil (189 mg, 41%).^1^H NMR (400 MHz, CDCl_3_) δ 7.44–7.15 (m, 5H), 3.93 (dd, *J* = 11.9, 7.5 Hz, 1H), 3.84 (dd, *J* = 11.9, 6.8 Hz, 1H), 2.23 (t, *J* = 7.1 Hz, 1H), 1.49 (s, 3H). ^13^C NMR (100 MHz, CDCl_3_) δ 155.48, 128.37, 128.34, 128.18, 127.16, 125.68, 59.81, 41.51, 38.22, 29.61, 24.56.

## Figures and Tables

**Table 1:** Dibromocyclopropanation of a selection of unsaturated alcohols using 40% (*w*/*w*) NaOH (aq) at AO ratio: 4.^a.^

Entry	Substrate	CHBr_3_ (eq) ^b^	Product		Yield (%)	Litt. Yield (%) ^c^	Reference
**1**	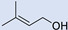	1.5	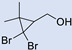	(**1**)	70 ^d^	36	[32]
**2**	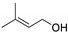	2	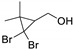	(**1**)	74 ^d^	36	[32]
**3**	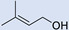	2.5	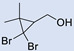	(**1**)	78 ^d^	36	[32]
**4**	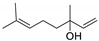	2	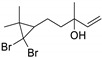	(**2**)	89 ^d^	93	[14]
**5**	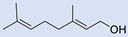	2	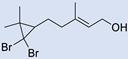	(**3**)	-^e^	-	[14]
**6**	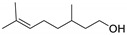	2.5^f^	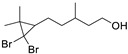	(**4**)	57 ^g^	-	-
**7**	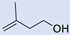	2	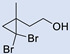	(**5**)	47 ^g^	58	[33]
			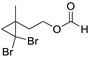	(**6)**	3 ^g^	-	-
**8**	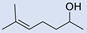	2	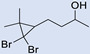	(**7**)	77 ^g^	-	-
**9**	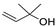	2	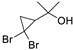	(**8**)	- ^h^	2	[34]
**10**	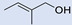	2	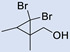	(**9**)	62 ^d,i^	45	[14]
**11**	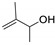	2	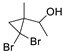	(**10**)	49 ^g,j^	62^k^	[14]
**12**	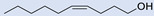	2	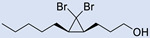	(**11**)^l^	- ^m^	-	-
**13**	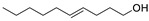	2	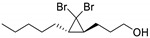	(**12**)^l^	- ^m^	-	-
**14**	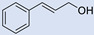	2	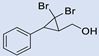	(**13**)	- ^m^	-	-
**15**	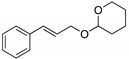	2	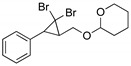	(**14**)	- ^m^	-	-
**16**	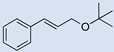	2	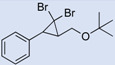	(**15**)	- ^m^	-	-
**17**	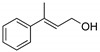	2	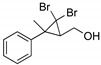	(**16**)	41 ^g^	-	-
**18**	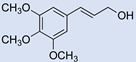	2	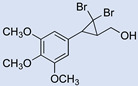	(**17**)	- ^m^	-	-
**19**	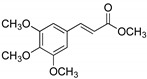	2	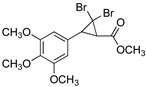	(**18**)	- ^m^	-	-

^a^ Conditions unless otherwise stated: [alkene] = 1.4 M, 4.2–4.3 moL% TEBA (relative to alkene), 0.6 vol% ethanol (in CH_2_Cl_2_). Room temperature, 25 mL PTFE tube reactor. Total flow rate of 0.50 mL/min. Aqueous to organic flow ratio (AO ratio): 4. Reaction time 50 min. ^b^ Relative to alkene. ^c^ Literature yields are only given for Makosza conditions using TEBA. ^d^ Estimated using ^1^H NMR spectra of the isolated reaction mixture. ^e^ Recovery of starting material not attempted (due to complexity of reaction mixture.) ^f^ With only two equivalents, the reaction did not go to completion. ^g^ Isolated yield. ^h^ Recovery of starting material not attempted (due to the low boiling point (< bp. of bromoform)). ^i^ 0,8% ethanol added. ^j^ No ethanol added. ^k^ Ratio of diastereoisomers 45:55. ^l^ As a racemate. ^m^ The substrate did not react, and >90% was recovered.
